# Bifunctional peptides as alternatives to copper-based formulations to control citrus canker

**DOI:** 10.1007/s00253-023-12908-3

**Published:** 2024-02-07

**Authors:** Guilherme Dilarri, Leticia Celia de Lencastre Novaes, Felix Jakob, Ulrich Schwaneberg, Henrique Ferreira

**Affiliations:** 1 Department of Fisheries Engineering and Biological Sciences, Santa Catarina State University (UDESC), Rua Coronel Fernandes Martins 270, Postal code, Laguna, SC 88790-000 Brazil; 2 DWI – Leibniz-Institute for Interactive Materials, Forckenbeckstraße 50, Postal code, 52056 Aachen, Germany; 3 Institute of Biotechnology, RWTH Aachen University, Worringerweg 3, Postal code, 52074 Aachen, Germany; 4 Institute of Biosciences, Biochemistry Building, Department of General and Applied Biology, State University of Sao Paulo (UNESP), Avenida 24-A 1515, Postal code, Rio Claro, SP 13506-900 Brazil

**Keywords:** Melittin, Citrus leaf-binding, Anchor peptide, Citriculture defensive, *Xanthomonas citri*

## Abstract

**Abstract:**

Citrus canker is an infectious bacterial disease and one of the major threats to the orange juice industry, a multibillion-dollar market that generates hundreds of thousands of jobs worldwide. This disease is caused by the Gram-negative bacterium *Xanthomonas citri* subsp. *citri*. In Brazil, the largest producer and exporter of concentrate orange juice, the control of citrus canker is exerted by integrated management practices, in which cupric solutions are intensively used in the orchards to refrain bacterial spreading. Copper ions accumulate and are as heavy metals toxic to the environment. Therefore, the aim of the present work was to evaluate bifunctional fusion proteins (BiFuProts) as novel and bio-/peptide-based alternatives to copper formulations to control citrus canker. BiFuProts are composed of an anchor peptide able to bind to citrus leaves, and an antimicrobial “killer” peptide to protect against bacterial infections of plants. The selected BiFuProt (Mel-CgDEF) was bactericidal against *X*. *citri* at 125 μg mL^−1^, targeting the bacterial cytoplasmic membrane within the first minutes of contact. The results in the greenhouse assays proved that Mel-CgDEF at 250 μg mL^−1^ provided protection against *X*. *citri* infection on the leaves, significantly reducing the number of lesions by area when compared with the controls. Overall, the present work showed that the BiFuProt Mel-CgDEF is a biobased and biodegradable possible alternative for substitute cupric formulations.

**Key points:**

*• The bifunctional fusion protein Mel-CgDEF was effective against Xanthomonas citri.*

*• Mel-CgDEF action mechanism was the disruption of the cytoplasmic membrane.*

*• Mel-CgDEF protected citrus leaves against citrus canker disease.*

**Supplementary Information:**

The online version contains supplementary material available at 10.1007/s00253-023-12908-3.

## Introduction

Citriculture is one of the most profitable commodities in the world, which generates hundreds of thousands of jobs in producing countries like Brazil, China, and the USA. Brazil and the USA are responsible for almost half of all the global sweet orange production (Neves et al. 2020). Brazilian production reached 294.17 million orange boxes of 40.8 kg during the 2021/2022 season, according to the Fund for Citrus Protection (Fundecitrus), an organization of Brazilian citrus producers and processors. Roughly, Brazil is responsible for 56% of orange juice and 35% of global fruit production according to estimates of the United States Department of Agriculture (USDA) (USDA 2015; Curtolo et al. 2017). Despite the great success, bacterial diseases impose constant threats to citriculture, leading to substantial yield losses in all major cultivation areas (Mendonça et al. 2017). Among the infectious diseases, citrus canker is one of the most relevant bacterial diseases, which affects all the commercially important citrus varieties worldwide (Gochez et al. 2020; Martins et al. 2020). The etiological agent of citrus canker is the Gram-negative bacterium *Xanthomonas citri* subsp. *citri* (Gottwald et al. 2002; Schaad et al. 2006). *X*. *citri* forms biofilms and remains firmly attached to fruits, as well as to all aerial plant parts, colonizing and infecting plants through natural openings (stomata) and wounds (Yaryura et al. 2015). *X*. *citri* spreads to other plants by the combined action of rain and wind (Ference et al. 2018), *Phyllocnistis citrella* (citrus leafminer) also increases the bacterial spread. Citrus leafminer is a pest that directly influences citrus canker, increasing the entry area of the bacteria in the leaves, and further spreading the disease (Mansour et al. 2021; Nawaz et al. 2021). Citrus canker is characterized by brownish erumpent lesions, and when the disease reaches high infection levels, it leads to plant defoliation and premature fruit drop, being responsible for decreased productivity (Gottwald et al. 2002; Behlau 2021). Moreover, contaminated fruit cannot be commercialized to avoid the spread of the bacterium to regions free of citrus canker (Ference et al. 2018; Zamuner et al. 2020).

The control of citrus canker is done by frequent sprays of metallic copper-based formulations, which can protect leaves from bacterial infection (Behlau et al. 2017). Cupric defensives show long-lasting protection because of the high content of metallic copper that deposits and forms films on the plant surfaces. Copper metal films exert mechanic barrier against pathogens, besides, the subsequent ionization of copper by water contact can also kill microorganisms due to its biocidal action. However, constant application of copper has the adverse effect of accumulating this heavy metal in the soil at toxic levels, which diminishes biodiversity, as well as affects important ecological relations in the environment (Martinez et al. 2016; Tóth et al. 2016). Once applied in the field, copper ions leach further, e.g., contaminating rivers, lakes, and the ocean, and affecting organisms in these aquatic environments as well (Soedarini et al. 2014). Recent studies have even linked the effect of the high application of copper in agriculture with Alzheimer’s disease in humans (Coelho et al. 2020). In addition, resistance to copper has already emerged in *X*. *citri* (Canteros et al. 1995).

Due to their toxicity to the environment and human health, several copper-based agrochemicals have their use restricted, or can only be applied in limited amounts (e.g., defined by the European Commission) (Lamichhane et al. 2018). Nevertheless, citrus canker is almost exclusively managed with copper formulations (Ference et al. 2018). Though copper is the bactericide of choice used in agriculture all over the world since 1800, the search for alternatives capable of minimizing or even avoiding the use of copper is of great interest for safer and more sustainable agriculture (Lamichhane et al. 2018).

Peptide-based pesticides are usually not accumulating in soil due to their biodegradability, act due to their natural design against specific organisms and are therefore not harmful to humans. In addition, antimicrobial peptides (AMPs) are effective in very small quantities and decompose quickly in the environment, e.g., through extracellular soil proteases (Vranova et al. 2013). AMPs are in summary promising candidates to be used as active ingredients in biopesticides. AMPs are small cationic peptides that protect their hosts against bacteria, protozoa, viruses, and fungi (Izadpanah and Gallo 2005; Guani-Guerra et al. 2010). These evolutionarily developed peptides, in general, constitute a highly heterogeneous group of molecules, but share common features, like the small size (20–100 amino acids (aa)) and amphiphilic or hydrophobic properties (Hassan et al. 2012). Since AMPs have both hydrophobic and hydrophilic parts, they are soluble in aqueous environments and have the ability to enter lipid-rich membranes (Izadpanah and Gallo 2005). More than 3000 AMPs have been identified (Li et al. 2021) and certain AMPs are reported to be effective agents in combating tumor cells (Oliveira et al. 2021), clinical bacterial pathogens (Picoli et al. 2017; Kazemzadeh-Narbata et al. 2021) as well as phytopathogens (Das et al. 2019). Currently, seven AMPs are allowed by the US Food and Drug Administration (FDA) for use in food market or in drug development for clinical use, with an increasing trend being the development and application of AMPs in the afore mentioned areas (Li et al. 2021; Datta and Roy 2021).

Melittin was discovered in the 1970s, and it is one of the most widely studied AMPs. Melittin is the principal component in the venom of the European honeybee *Apis mellifera*, comprising at least 50% of the venom’s dry weight (Hong et al. 2019; Memariani et al. 2019). Melittin is a small cationic peptide, composed of 26 amino acid residues (GIGAVLKVLTTGLPALISWIKRKRQQ). The polar and nonpolar residues are asymmetrically distributed, suggesting its amphipathic nature when melittin adopted an α-helical conformation. This feature makes the peptide not only water-soluble but also membrane active (Memariani et al. 2019). The hydrophobic N-terminus and hydrophilic C-terminus are responsible for melittin’s characteristic structure of membrane-bound cytolytic and trans-membrane helices (Chen et al. 2016). Melittin has the ability to form pores across the lipid bilayer, which induces leakage, and at higher concentrations, membrane fragmentation (Shi et al. 2016; Therrien et al. 2016). Melittin has various biological, pharmacological, and toxicological actions, including strong surface activity on cell lipid membranes, antibacterial and antifungal activities, anti-inflammatory, and potential anti-tumor properties (Chen et al. 2016; Therrien et al. 2016). Although melittin has been extensively studied regarding its antibacterial activity, there are only a few reports about its action against plant pathogens. Shi et al. (2016) reported that melittin was active against *Xanthomonas oryzae* pathovar *oryzae*, the etiological agent of rice blight disease. Due to its quick effect, characterized by membrane disruption, resistance to melittin is difficult to be developed. Main limitation for broader use of AMPs in plant health is that as many (bio-)pesticides AMPs are easily washed off from plant leaves by rain events.

Anchor peptides are adhesion promoting peptides that can be engineered to bind to natural (plant leaves, teeth) (Meurer et al. 2017; Schwinges et al. 2019) and synthetic surfaces (polymers), including metals (Apitius et al. 2019; Dedisch et al. 2020; Nöth et al. 2020). Tailor-made anchor peptides are thus applicable in many fields, including biotechnology, catalysis, medicine, and plant health (Meurer et al. 2017; Gao et al. 2018; Grimm et al. 2019). In a first previous study by our group, anchor peptides were genetically fused to an anti-fungal peptide. This fusion peptide successfully protected soybean plants against its most severe disease, the Asian soybean rust (caused by *Phakopsora pachyrhizi*) in a rain fasten manner (Schwinges et al. 2019). These so-called bifunctional peptides (BiFuProts) consist of one peptide that adheres to epicuticular surface waxes of the leaves (responsible for strong rain fastness) while the other peptide possessed antimicrobial activity against *P. pachyrhizi*.

To provide a sustainable plant health solution for a heavy metal-free production of oranges, we designed a bifunctional peptide design principle, which protects plants against an important bacterial plant pathogen (*X. citri*). Within this study, we report for the first time the successful protection of citrus plants against *X. citri* by a bifunctional fusion protein applied through spraying.

## Material and methods

### eGFP and eGFP-peptide fusion

Six different peptides (Androctonin (Andt), defensin (CgDEF), liquid chromatography peak I (LCI), antimicrobial peptide MBP-1 (MBP1), plantaricin A (PlnA), tachystatin A2 (TA2)), and the antimicrobial peptide melittin (Mel) were genetically fused to the reporter protein eGFP (enhanced green fluorescent protein) to investigate their ability to bind to the surface of orange leaves. The respective amino acid sequences are listed in Table S1 from the Supplementary Material. eGFP-peptide fusions consisted of N-terminal His_6_-eGFP and a C-terminal peptide. The C-terminal peptide was separated via a stiff helical spacer consisting of 17 amino acids (AEAAAKEAAAKEAAAKA) (Arai et al. 2001) followed by a tobacco etch virus (TEV) protease cleavage site of 7 amino acids (ENLYFQG) from eGFP (Kapust et al. 2001). Generation, production, and purification were performed as described elsewhere (Schwinges et al. 2019). The analysis of eGFP (negative control for background binding) and the fusion proteins eGFP-Andt, eGFP-CgDEF, eGFP-LCI, eGFP-MBP1, eGFP-PlnA, eGFP-TA2, and eGFP-Mel was executed similarly as described elsewhere (Schwinges et al. 2019). Leaf disks (~1 cm diameter) of orange leaves (*Citrus sinensis*), which had an average size of ~4 cm, were used for the binding tests. The disks were immersed in cell free *Escherichia coli* BL21 (DE3) (purchased from Agilent Technologies Inc., Santa Clara, USA) lysates containing eGFP, eGFP-Andt, eGFP-CgDEF, eGFP-LCI, eGFP-MBP1, eGFP-PlnA, eGFP-TA2, or eGFP-Mel in separated wells of a microtiter plate. After 5 min, the leaf surface was washed by rinsing with Tris/HCl buffer (50 mM pH 8.0), and the surface was analyzed using confocal microscopy (Leica TCS SP8 microscope, Ex 485 nm, Em 520 nm, argon laser 20% intensity, gain 500, Leica Microsystems GmbH (Wetzlar, Germany)).

### BifFuProt design and production

The generated BiFuProt consists of the antimicrobial peptide melittin and the orange leaf binding peptide CgDEF (Mel-CgDEF). The synthetic gene (GenScrip Biotech, Rijswijk, Netherlands) was ordered within the pET28a(+) expression vector, which was transformed into *Escherichia coli* BL21 (DE3) cells. The BiFuProt consisted of an N-terminal melittin that was linked via a flexible 3xGGGS sequence and a StrepII-tag with domain Z. Domain Z separates the two functional peptides from each other in order to minimize intramolecular interactions. The anchor peptide CgDEF was fused to the C-terminus of domain Z (the amino acid and DNA sequence of the bifunctional peptide is given in Table S2 from the Supplementary Material). A single colony was transferred to 5 mL of LB medium and incubated (16 h, 37 °C, 180 rpm; Multitron Pro, Infors AG, Bottmingen, Switzerland). The pre-culture (1 mL) was used to inoculate the main culture (100 mL TB, 37 °C, 180 rpm). The main culture was cultivated until the OD_600nm_ reached 0.6. Protein over-expression was induced by supplementing IPTG (isopropyl-β-D-thiogalactopyranoside) to a final concentration of 0.1 mM. Upon induction, the cultivation temperature was reduced to 20 °C. Cells were harvested after 24 h by centrifugation (3200 ×*g*, 20 min, 4 °C) and the cell pellets were stored at −20 °C until further processing or immediately used for cell disruption. Cell disruption was performed by sonication. The cell pellet was resuspended in Tris-HCl buffer (50 mM, pH 8.0) in the ratio of 4 mL of buffer per gram of cells. Sonication was performed on ice (Vibra-Cell VCX 130; VWR International, Radnor, Pennsylvania, USA; amplitude of 70% in 2 cycles of 2 min, pulse of 10 s ON/10 s OFF). Cell debris was removed through centrifugation (3200 ×*g*, 45 min, 4 °C) and the supernatant was filtered through a 0.45 μm cellulose-acetate filter (GE Healthcare, Chalfont St Giles , UK) and subsequently used for protein purification. The construct contains a StrepII-Tag and was purified using a fast protein liquid chromatography system (ÄKTAprime, GE Healthcare, Chalfont St Giles, UK) with a prepacked Strep-Tactin affinity chromatography column (5 mL, Strep-Tactin Superflow Plus Cartridges, Qiagen, Hilden Germany). Elution was performed with 50 mM NaH_2_PO_4_.2H_2_O, 300 mM NaCl, and 2.5 mM desthiobiotin. For the plant protection assay bifunctional peptides were purified using a fast protein liquid chromatography system (ÄKTAprime, GE Healthcare, Chalfont St Giles, UK) with a prepacked cation exchange chromatography column (HiTrap SP FF Cartridges, 5 mL, GE Healthcare, Chalfont St Giles, UK ) and gradual elution (45 to 100%) with phosphate buffer 50 mM pH 6.0 + 1 M NaCl. After purification, samples were desalted using a dialysis membrane (Spectra/Por4, Spectrum Inc., Breda, Netherlands) and stored at 4 °C until further use.

### Cultivation of the phytopathogen *X. citri*

The *X*. *citri* strain used was the isolate 306 - IBSBF 1594 (Schaad et al. 2006). The bacterium was cultivated in solid and liquid NYG medium (nitrogen, yeast extract, and glycerol: 5 g L^−1^ of peptone, 3 g L^−1^ of yeast extract, 2% glycerol; for solid medium bacterial agar was added to 15 g L^−1^) at 29 ± 1 °C with constant agitation at 200 rpm for liquid growth.

### Peptide inhibition assay

The inhibitory concentrations (IC) of the peptides were determined using the Resazurin Microtiter Assay Plate method (REMA) with some adaptations as described by Silva et al. (2013). Briefly, the purified peptide diluted in NYG medium were added to the wells of a 96-microtiter plate to obtain the final concentrations of 250, 125, 62.5, 31.25, 15.62, 7.81, 3.90, and 1.95 μg mL^−1^ in total volumes of 100 μL per well. The positive control was kanamycin at 20 μg mL^−1^, the negative control was NYG medium, and the vehicle control was constituted of 50 μL of NYG medium and 50 μL of deionized water. *X*. *citri* was then inoculated in each well, with a final concentration of 10^5^ CFU (colony-forming unit) per well. REMA plates were incubated at 29 ± 1 °C for 16 h. To develop the reactions resazurin (Sigma-Aldrich, Darmstadt, Germany) was added to each well to the final concentration of 0.01 mg mL^−1^, and plates were further incubated at 29 ± 1 °C for 2 h. NADH produced by the live/respiring cells reduces resazurin to the fluorescent compound resorufin, which was detected using a plate reader Synergy H1N1 (BioTek, Winooski, VT, USA) set to the excitation and emission wavelengths of 530 and 590 nm, respectively. The data obtained were used to construct dose-response curves, and a polynomial regression model was applied in order to determine the growth inhibition percentages expressed as the “inhibition concentration values” (IC), which corresponds to the peptide concentration able to inhibit a certain percentage of *X*. *citri* cells in vitro. To define the minimal bactericidal concentration (MBC), samples from REMA were inoculated on a solid NYG medium, using 15 cm Petri dishes, with the help of a 96-well plate replicator (8 × 12 wells, Sigma-Aldrich Corp., St. Louis, USA). The plates were then incubated at 29 °C for 48 h to allow bacteria to resume growth. Three independent experiments were performed in triplicates.

### Fluorescence microscopy

*X*. *citri* cells (10^5^ CFU mL^−1^) were exposed to the bifunctional peptide at its bactericidal concentration for 15 min using 1.5 mL microcentrifuge tubes in a total volume of 100 μL of NYG medium. After the treatment, 900 μL of saline solution (0.86% NaCl) were added to the tubes in order to dilute the compound and stop the reaction. The viability of bacterial cells and membrane integrity were evaluated by using the live/dead method, in which cells were stained with propidium iodide (PI) at 10 μg mL^−1^ and 4′,6-diamidino-2-phenylin-dole (DAPI) at 20 μg mL^−1^. Untreated cells were used as negative control, while the positive control for membrane damage was obtained by heat-shock stress (Savietto et al. 2018). The *X*. *citri* mutant expressing GFP-ZapA (*X. citri amy*::pPM2a-*zapA*) was used to evaluate if the bifunctional peptide could interfere with the assembly of the bacterial divisional septum (defined as the Z-ring) (Silva et al. 2013). First, bacterial cells were cultivated in the presence of 0.5% xylose to induce the expression of GFP-ZapA (Martins et al. 2010). Next, 100 μL of the culture containing 10^5^ CFU mL^−1^ were placed in a 1.5-mL microcentrifuge tube and exposed to the peptide at its bactericidal concentration for 15 min. Then, the reaction was centrifuged at 4000 × *g* for 5 min, the supernatant was discarded, and the cells were resuspended in 100 μL of saline solution (0.86% NaCl). Regarding the visualizations, cells were immobilized onto agarose-covered slides and subsequently observed using a fluorescence microscope Olympus BX-61 (Tokyo, Japan), equipped with a monochromatic camera OrcaFlash 2.8 (Hamamatsu, Shizuoka, Japan). Images were obtained and processed using the software CellSens Dimension (Olympus, Tokyo, Japan). Three independent experiments were performed, and at least 100 cells were considered per treatment (*n* = 100) for the analyses.

### Plant protection assays

The ability of the bifunctional peptide to protect citrus leaves against *X*. *citri* infection was evaluated using seedlings of *C*. *sinensis* cultivar Pera of ~50 cm tall. The plants were kept in a greenhouse with controlled humidity and temperature as follows: average humidity of 77.90% with a maximum of 92.70% and a minimum of 50.27%; average temperature of 26.36 °C, with a maximum of 37.54 °C and a minimum of 17.84 °C (during all the experiment period from 06/11/2020 to 22/01/2021). Different peptide concentrations, ranging from 62.50 to 250 μg mL^−1^, were sprayed on leaves until the run-off point. For the positive control, the plants were sprayed with a commercial copper formulation (Difere® at the recommended concentration of 2.96 mg mL^−1^, 700 μg mL^−1^ of metallic copper)—Oxiquímica Agrociência Ltda., Jaboticabal, Brazil. The negative control was saline solution (0.86% NaCl). After 24 h, the plants were sprayed with a 10^8^ CFU mL^−1^ suspension of *X*. *citri* in saline solution (0.86% NaCl). After inoculation, plants were covered with transparent plastic bags for 24 h to help bacterial infection. Plants were observed over the course of 35 days for the appearance of symptoms. The experiment was performed with two plants per group of treatment, with two marked branches per plant. This assay was done with three independent replicates. At the end of the experiment, leaves were detached and digitalized at 600 dpi. The software ImageJ (Fiji) (Rasband 2018) was used to evaluate bacterial infection and infection severity, expressed by the number of citrus canker lesions formed per leaf area, as described by Cavalca et al. (2020). The data obtained were submitted to a nonparametric statistical analysis of Kruskal-Wallis (Dunn), with three degrees of freedom using the software BioEstat 5.0 (Ayres 1998).

To verify if the bifunctional peptide could remain fixed on the leaf surface the experiment above was repeated with the addition of a washing step in order to simulate rain. Marked branches were sprayed until the run-off point with each treatment as follows: I) saline solution (0.86% NaCl) as the negative control, II) 2.96 mg mL^−1^ of Difere® as the positive control, and III) concentrated peptide solution. Twenty-four hours later, plants were sprayed with 100 mL of autoclaved deionized water until the run-off point (approximately 2 min). After the wash step, plants were sprayed with the inoculum (*X*. *citri* at 10^8^ CFU mL^−1^) in saline solution (0.86% NaCl) and then covered with transparent plastic bags for further 24 h. The appearance of symptoms was monitored for 35 days. It was used again two plants per treatment with two marked branches per plant, and the experiment was carried out with three independent replicates. The protective efficacy was measured as described above by determining the number of lesions formed per leaf area.

## Results

### Peptide binding to orange leaves

**Fig. 1:**
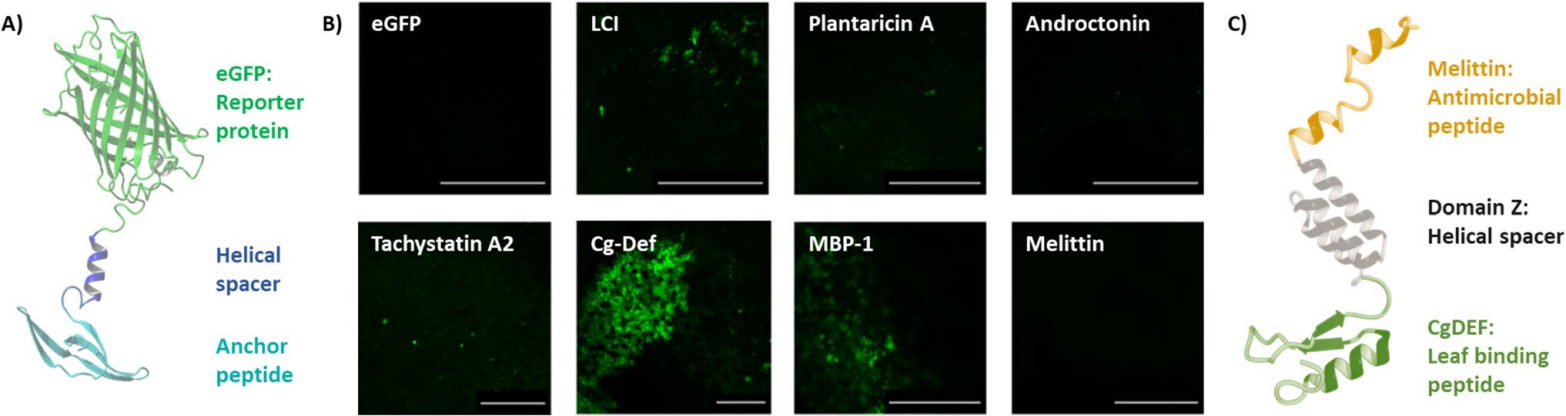
Result of binding peptide assays. **A** Schematic representation of anchor peptides that are fused to the reporter protein eGFP (enhanced green fluorescent protein) separated by a stiff helical linker. These fusion proteins were used to investigate peptide binding to orange leaves. **B** Fluorescence confocal microscopy visualization of eGFP (top-left), eGFP-anchor peptides, and eGFP-Melittin (bottom-right) bound to the surface of orange leaf disks after a washing event. **C** Schematic representation of the bifunctional protein Mel-CgDEF. The BiFuProt consisted of an N-terminal melittin (PDB: 2MLT) that was linked via a flexible 3xGGGS sequence (not shown) and a StrepII-tag (not shown) with domain Z (PDB: 2B88). Domain Z separates the two functional peptides from each other in order to minimize intramolecular interactions. The anchor peptide Cg-DEF (PDB: 2B68) was fused to the C-terminus of domain Z. A 3D model generated by AlphaFold 2.0 (Jumper et al. 2021) is shown in Fig. S2 from the Supplementary Material

To investigate the ability of the anchor peptides to bind to orange leaves, selected peptides were fused to eGFP in order to enable their easy detection on the surface of orange leaves using fluorescent microscopy. eGFP-LCI, eGFP-CgDEF, and eGFP-MBP1 were able to bind to the orange leaf surface in a washing-resistant manner (Fig. 1). On the other hand, the anchor peptide eGFP-TachA2 was weak in binding on the orange leaf surface, as shown in Fig. 1. The control eGFP, as well as eGFP-Mel, eGFP-PlnA, and eGFP-Andt, did not show a significant fluorescence on the leaf surface after a washing event. The densest coverage of the leaf surface was achieved by the eGFP-CgDEF; therefore, this anchor peptide was selected to generate the bifunctional fusion protein described below.

In a similar past work done by Schwinges et al. (2019), a BiFuProt was constructed to inhibit the infection of soybean rust, with the anchor peptides TachA2 and LCI. However, in the binding study with the orange leaves, the TachA2 and LCI did not show a high leaf fixation as CgDEF. It is two different genera of plants, therefore is expected that different anchor peptides will get different fixation interactions with the leaf surfaces. So, even with the similarity with the work described by Schwinges et al. (2019), the most suitable protein construction for binding in orange leaves is using the CgDEF peptide as an anchor.

### Bifunctional fusion protein exhibited anti-*X. citri* activity

**Fig. 2:**
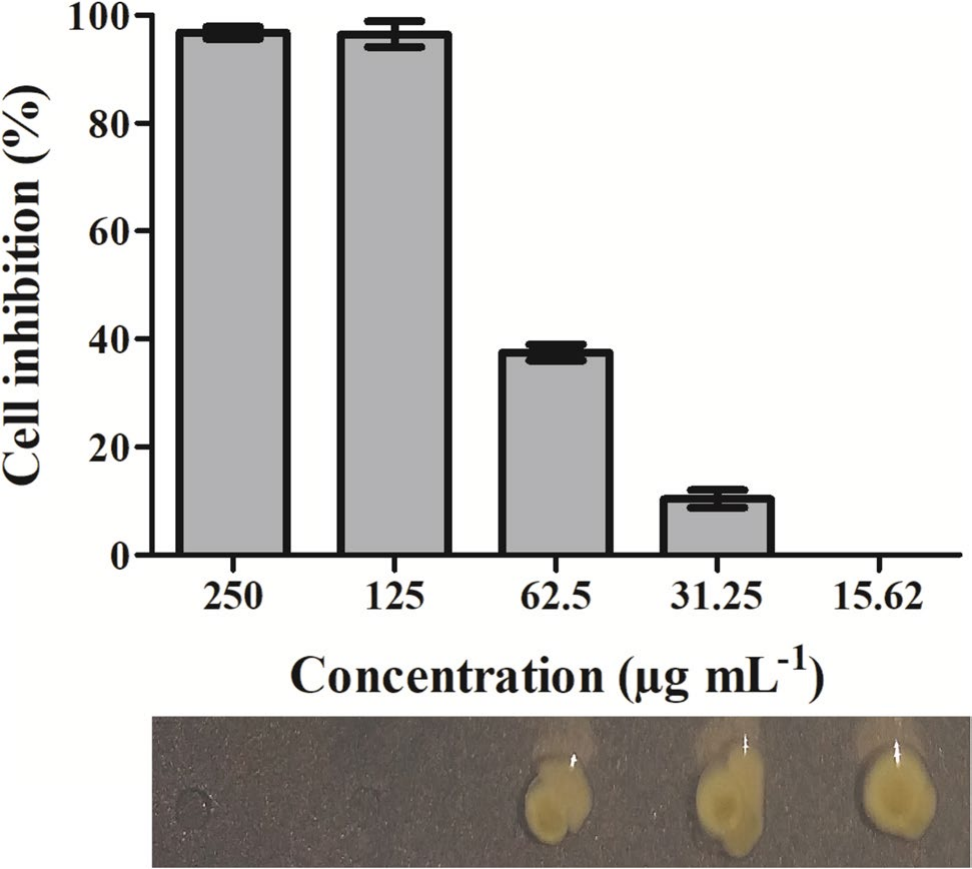
Mel-CgDEF has bactericidal activity against *X. citri*. The respiratory activity of the cells was monitored by using resazurin after 16 h of peptide exposure. The average percentages of cells inhibited by the peptide are represented as bars and the standard deviation of the means by the vertical lines above the bars. Underneath the graph is shown a section of a NYG-plate in which samples from REMA were inoculated in order to evaluate if Mel-CgDEF had bactericidal effect. Yellow colonies indicate growth

The causal agent of citrus canker, the bacterium *X*. *citri*, was exposed to different concentrations of Mel-CgDEF, and the respiratory activity of the cells was monitored using REMA. The peptide was able to inhibit *X*. *citri* growth with clear dose-response behavior (Fig. 2). Non-linear regression was subsequently used to estimate inhibitory concentrations (IC values) of Mel-CgDEF against *X*. *citri*. In such analysis, 125 μg mL^−1^ of Mel-CgDEF was defined as the concentration of the peptide able to completely inhibit bacterial growth after 16 h of exposure. In order to determine the minimal bactericidal concentration of Mel-CgDEF, aliquots from REMA were spread onto a solid NYG medium before resazurin was added. Cells treated with the peptide at the IC (125 μg mL^−1^) were not able to resume growth even after 72 h of incubation (Fig. 2). Therefore, Mel-CgDEF was considered bactericidal when used at 125 μg mL^−1^.

To certify and validate which part of the BiFuProt Mel-CgDEF was responsible for the anti-*X. citri* activity observed above, the modules eGFP-CgDEF, eGFP-Mel, and Mel-eGFP were evaluated in REMA independently against the bacterium. The fusion eGFP-CgDEF did not show any inhibitory effect against *X. citri*, while the eGFP-Mel (melittin at the C-terminus of eGFP) showed a bactericidal effect at 76.80 μg mL^−1^ (Fig. S1 from the Supplementary Material). Mel-eGFP (melittin at the N-terminus of eGFP) also exhibited bactericidal action against *X. citri* at a concentration of 72.10 μg mL^−1^ (Fig. S1 from the Supplementary Material). Since the position of the antimicrobial peptide with respect to eGFP did not significantly influence its antimicrobial activity, we decided to fuse melittin to the N-terminus of CgDEF within the bifunctional fusion protein for further studies.

### Cytoplasmic membrane is the primary target of Mel-CgDEF

**Fig. 3:**
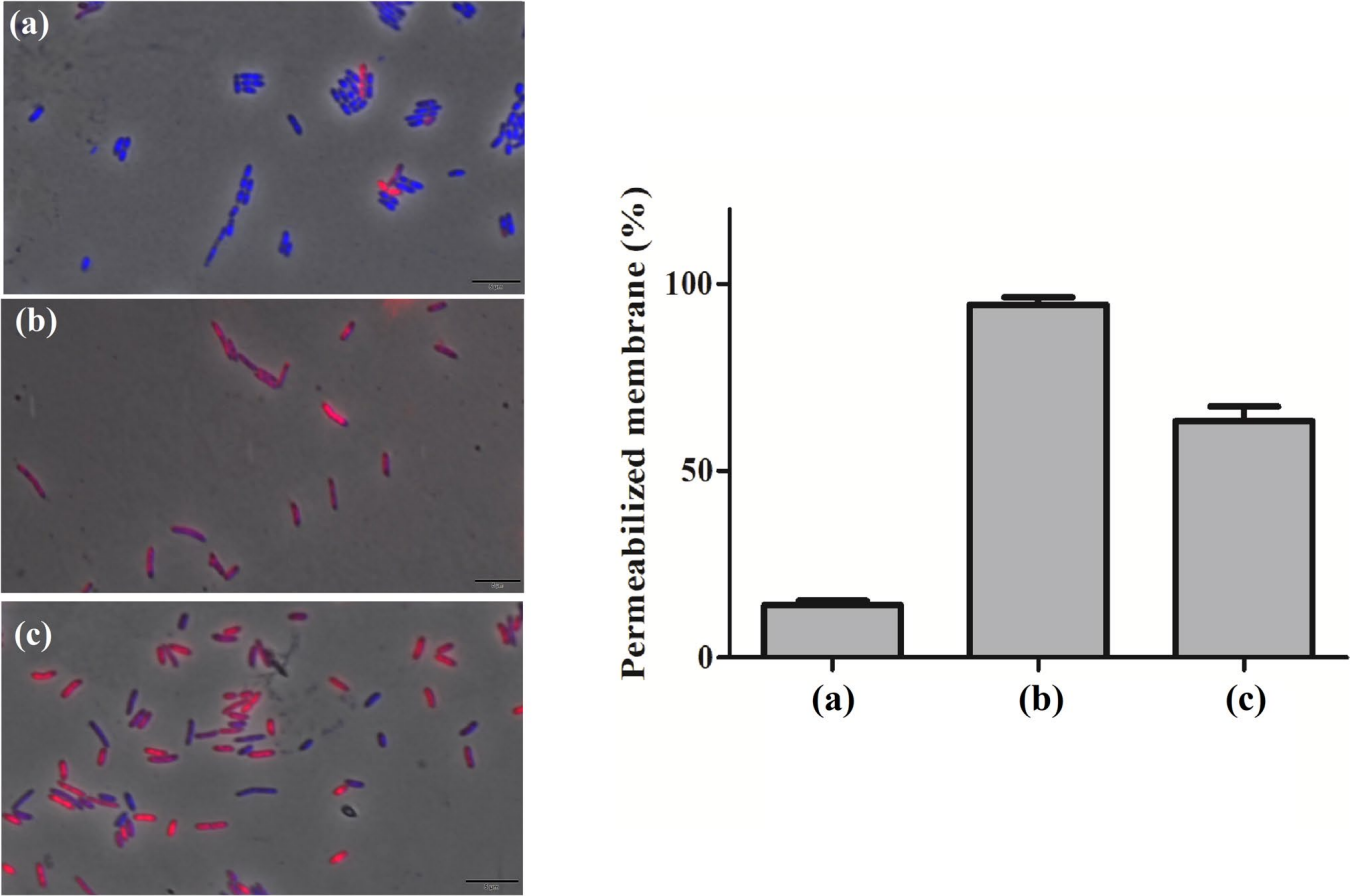
The cytoplasmic membrane is the primary target of Mel-CgDEF. Cells of *X*. *citri* were exposed to the peptide for 15 min, and after stained with DAPI and PI. Cells with intact membranes are represented in blue, while the cells with damaged membranes are colored in red. **a** Negative control, untreated *X*. *citri* cells; **b** positive control, cells subjected to thermal stress; **c** *X*. *citri* cells after 15 min of contact with Mel-CgDEF at 125 μg mL^−1^. Pictures show overlays of phase contrast and DAPI/PI images. Scale bar 5 μm; magnification of ×100. Bars represent the percentage of *X*. *citri* cells with permeabilized membrane after 15 min of contact with 125 μg mL^−1^ Mel-CgDEF

To investigate some of the possible targets of Mel-CgDEF in *X*. *citri*, we used a combination of phase contrast and fluorescence microscopy to check for membrane permeability and cell division alterations. Cells were exposed to the peptide for 15 min, which is considered a quarter of the doubling time for *X*. *citri*, and after stained with DAPI/PI for microscope observations (the nucleic acid dye DAPI penetrates all the cells; PI penetrates only of those cells with damaged membranes). Untreated *X*. *citri*, cultivated in a rich NYG medium, normally has 1.5 to 2 μm in length (a size considered standard; Fig. 3a). Moreover, we did not observe any filamentation, which could be an indication of interference with the cell division machinery. Cells here are clearly division competent with dividing rods displaying normal septal constrictions. Finally, normally growing *X*. *citri* may exhibit a small number of PI-permeable cells, usually less than 5% of the individuals in a culture (Fig. 3a; red-colored cells). When *X. citri* was exposed to Mel-CgDEF, cells still had a fairly normal morphology under phase contrast microscopy (Fig. 3c). Cells also kept the standard size of ~1.5 to 2 μm. In addition, we did not detect any cell filamentation as compared to the untreated cells (Fig. 3a). However, more than 60% of the cells after 15 min of peptide exposure were permeable to PI. Statistics of nearly 300 cells (three independent experiments with *n* = 100 per experiment) showed that on average 63.37% of cells had damaged membranes after 15 min of contact with Mel-CgDEF (Fig. 3c).

**Fig. 4:**
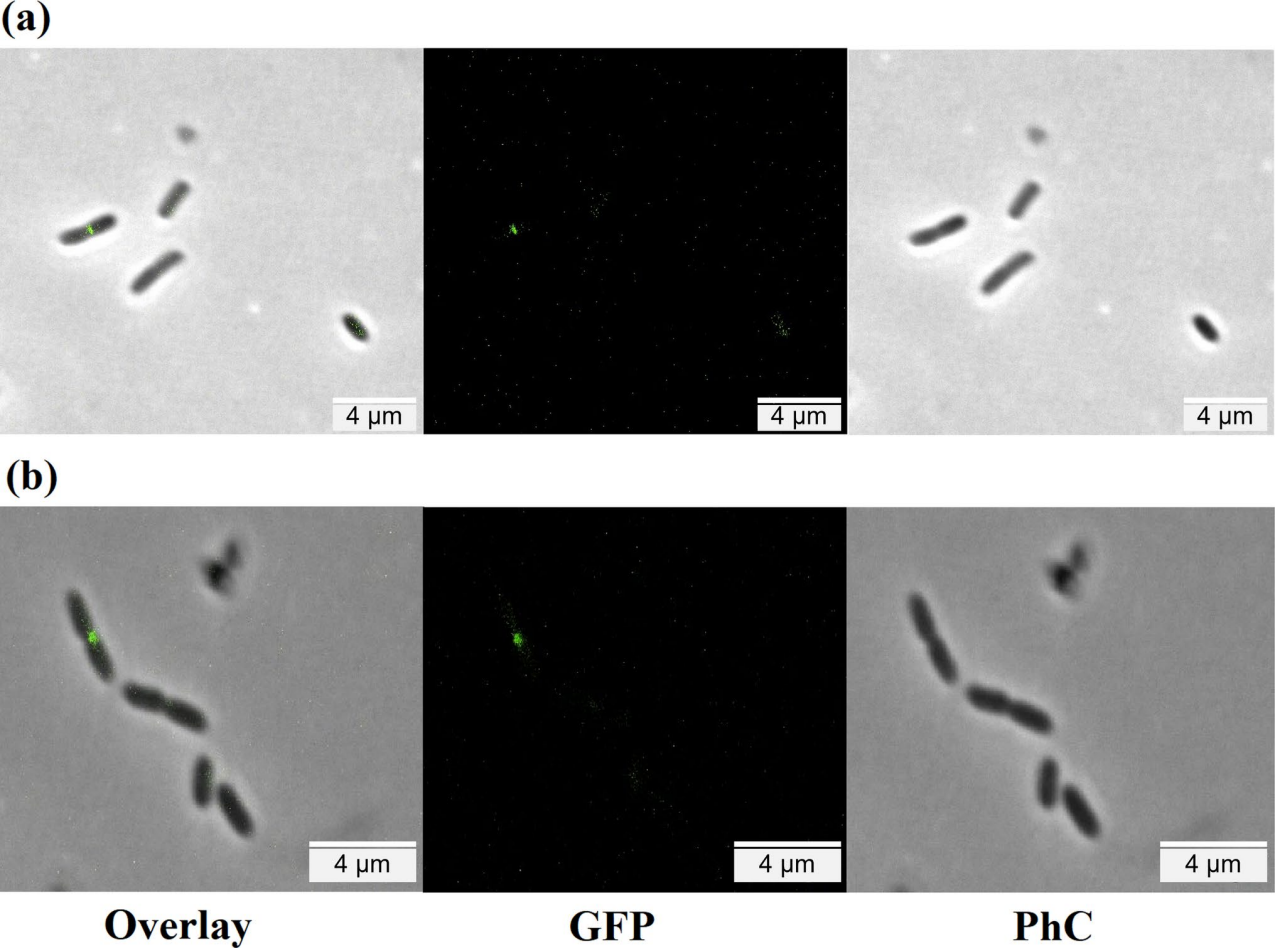
Mel-CgDEF does not target the bacterial divisome. The mutant strain *X*. *citri amy*::pPM2a-*zapA*, labelled for the divisional septum, was exposed to 125 μg mL^−1^ Mel-CgDEF for 15 min. **a** Untreated cells; **b** 125 μg mL^−1^ Mel-CgDEF. The divisional septum corresponds to the green bar perpendicular to the long axis of the rods. The scale bar corresponds to 2 μm; magnification of ×100, PhC is the phase contrast microscope configuration. A number of 300 cells (three independent experiments with *n* = 100 per experiment) were used as the total number of cells observed

We also investigated the action of Mel-CgDEF on the formation of the Z-ring itself. For that, we used the mutant *X*. *citri amy*::pPM2a-*zapA*, which expresses GFP-ZapA (a label for the Z-ring). *X*. *citri amy*::pPM2a-*zapA* was exposed to Mel-CgDEF at its bactericidal concentration and for the same time period used in the membrane integrity assays above-mentioned. As a result, we did not detect any visual alteration in the assembly of the Z-ring after the contact with the peptide (Fig. 4). In both panels (Fig. 4a, b; untreated cells and peptide exposed cells, respectively), we could detect the formation of the bacterial septum in dividing rods. Moreover, and similarly to when we used the wild type strain, no filamentation or gross morphology alterations were detected for the strain *X*. *citri amy*::pPM2a-*zapA* after exposure to the peptide (Fig. 4). Therefore, we conclude that the primary target of Mel-CgDEF is the cytoplasmic membrane of *X*. *citri*.

### The bifunctional fusion protein Mel-CgDEF protects citrus against *X. citri*

**Fig. 5:**
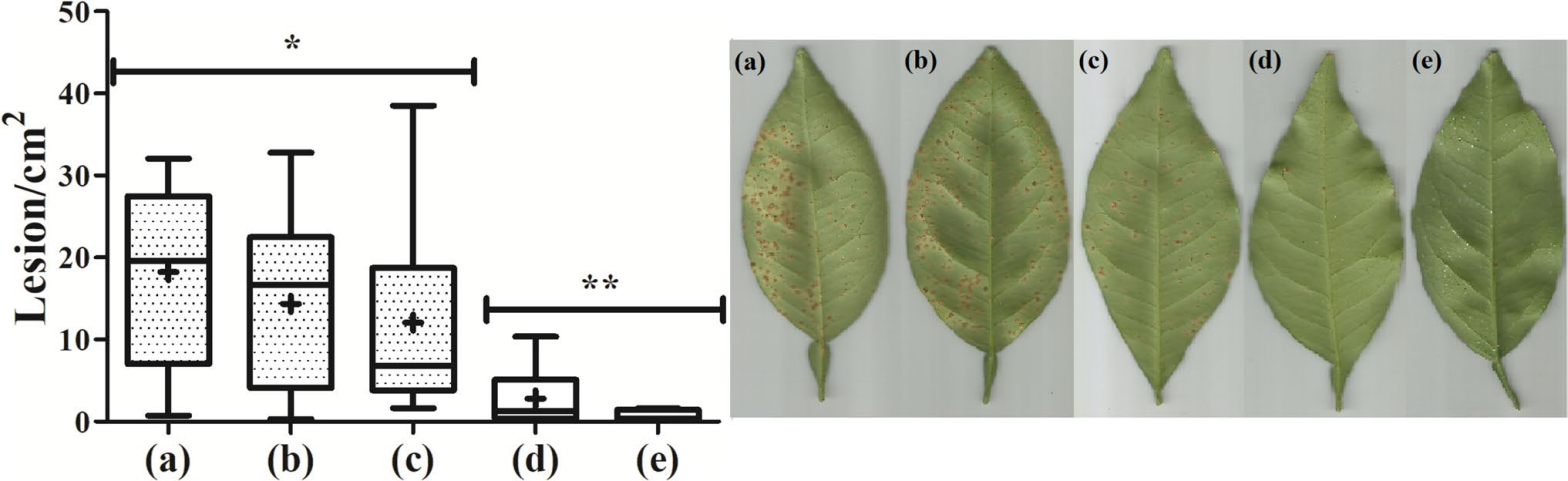
The BiFuProt Mel-CgDEF protects citrus against *X*. *citri* infection. Citrus seedlings were spray-covered with Mel-CgDEF or copper, and 24 h later challenged with the bacterium to evaluate the protective potential of the peptide. **a** Negative control, **b** Mel-CgDEF at 62.5 μg mL^−1^, **c** Mel-CgDEF at 125 μg mL^−1^, **d** Mel-CgDEF at 250 μg mL^−1^, **e** Difere® at 2.96 mg mL^−1^ (700 μg mL^−1^ of metallic copper). Boxes represent the distribution of 50% of the data per treatment; lines above and below the boxes correspond to the minimum and maximum values scored in the data sets; plus signs are the averages calculated for each distribution. Asterisks show the statistical difference among the treatments based on the Kruskal–Wallis Dunn analysis. On the right-hand side are illustrative pictures of leaves detached from the test plants

The ability of Mel-CgDEF to protect citrus against *X. citri* infection was evaluated using the seedling spray test. Plants were spray-covered with different concentrations of the BiFuProt, as well as the positive control for protection (the copper-based formulation Difere®), prior to bacterial challenge. Twenty-four hours later, bacteria were inoculated by spray until the run-off point, and two parameters were monitored in the course of the analyses: the ability to infect, which would lead to the appearance of citrus canker symptoms, and the severity of the infection (number of lesions/cm^2^ of leaf). Untreated leaves showed on average 18.22 lesion/cm^2^ (Fig. 5a). Leaves sprayed with Mel-CgDEF apparently showed increasing levels of protection as the concentration of peptide increased. This can be seen by the decreasing number of lesions formed per cm^2^ of leaf in the treatments with Mel-CgDEF at 62.50 μg mL^−1^ (Fig. 5b), 125 μg mL^−1^ (Fig. 5c), and 250 μg mL^−1^ (Fig. 5d), which were 14.37 lesion/cm^2^, 12.06 lesion/cm^2^, and 2.82 lesion/cm^2^, respectively. The protection level exerted by 250 μg mL^−1^ Mel-CgDEF was comparable and statistically equal to the positive control Difere® (Fig. 5e), which showed only 1.31 lesion/cm^2^. Even though the peptide showed some degree of protection when used at 62.50 μg mL^−1^ and 125 μg mL^−1^, only Difere® and Mel-CgDEF at 250 μg mL^−1^ were considered significantly different from the negative control untreated (18.22 lesion/cm^2^). Both, Difere® and Mel-CgDEF at 250 μg mL^−1^ could efficiently protect citrus plants against infection by *X. citri* (Fig. 5, compare d and e with a).

**Fig. 6:**
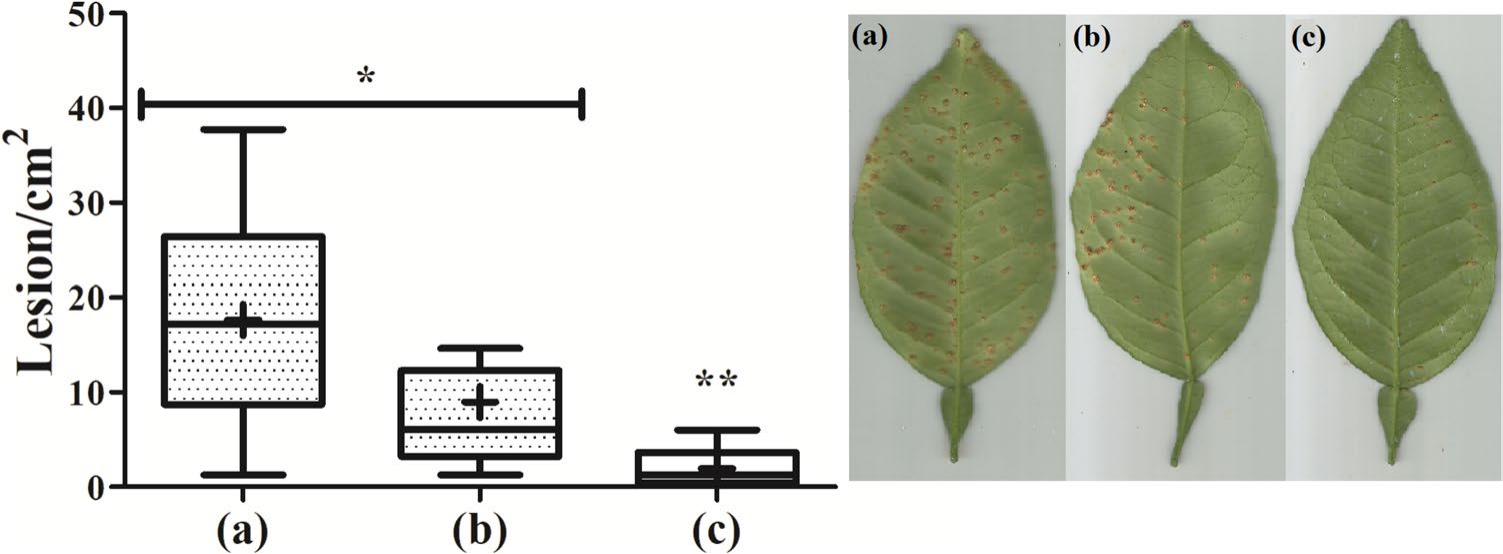
Rain fastness of Mel-CgDEF. Citrus seedlings were spray-covered with Mel-CgDEF or copper, and 24 h later washed with deionized water in order to simulate rain prior to bacterial challenge. **a** Negative control, **b** Mel-CgDEF at 250 μg mL^−1^, **c** Difere® at 2.96 mg/mL (700 μg mL^−1^ of metallic copper). Boxes represent the distribution of 50% of the data per treatment; lines above and below the boxes correspond to the minimum and maximum values scored in the data sets; plus signs are the averages calculated for each distribution. Asterisks show the statistical difference between the treatments based on the Kruskal–Wallis Dunn analysis. On the right side are illustrative pictures of leaves detached from the test plants

The leaf-binding peptide CgDEF fused to the C-terminus of the antimicrobial peptide melittin was originally selected based on its ability to bind and remain attached to citrus leaves. To verify the leaf retention potential of the BiFuProt Mel-CgDEF, and its protective efficacy after washing, leaves of citrus were spray-covered with the peptide, essentially as described above, and 24 h later washed with deionized water until the run-off point before the bacterial challenge. The Mel-CgDEF at 250 μg mL^−1^ reached 8.95 lesions/cm^2^, while the negative control showed an average of 17.63 lesions/cm^2^, being both statically equal (Fig. 6, compare a and b). The copper-based formulation, which is known to form films on the leaf surface, showed the same protective capacity as documented without washing (Fig. 6c; an average of 1.92 lesion/cm^2^). These greenhouse studies also showed that the treatment with melittin did not affect the plant height, tillering ability, leaf color, and shape, indicating nontoxic effects on orange trees.

## Discussion

Since the discovery of the Bordeaux mixture, copper is used in agriculture to control several infectious diseases (Lamichhane et al. 2018). In special, insoluble forms of copper are by far the most attractive and inexpensive plant defensives in use. Upon application of metallic copper on plant surfaces, they are known to form long-lasting films, which exert mechanic protection, as well as exhibiting microbicidal action against fungi and bacteria, by the subsequent slow and continuous solubilization in water (Ference et al. 2018; Lamichhane et al. 2018). Unfortunately, the extensively explored protective action of copper has led to its accumulation in soils, and water reservoirs, which can potentially cause toxicity to plants, animals, and also to the microbiota (Behlau et al. 2012; Behlau 2021). Although being considered an essential nutrient, exposure to copper and subsequent metal accumulation has been associated with Alzheimer’s disease (Coelho et al. 2020). Finally, tolerance and resistance to metals, including copper, in several etiological agents of infectious diseases has been documented, which impels us to consider more sustainable and less pollutant alternatives for the use of metals in agriculture (Behlau et al. 2012; Liu et al. 2017; Glibota et al. 2019; Behlau et al. 2020). A possible alternative explored in the present work was the use of antimicrobial peptides (AMPs) to protect citrus plants against bacterial infection. AMPs are small cationic peptides that protect their hosts against the attack by other microorganisms and viruses (Izadpanah and Gallo 2005; Guani-Guerra et al. 2010). We showed here that the bifunctional fusion protein (BiFuProt) Mel-CgDEF was active against *X. citri* inhibiting cell respiration as monitored by the redox agent resazurin. Mel-CgDEF was bactericidal against *X. citri* at 125 μg mL^−1^, which does not differ significantly from the action range of the standard/commercial antibiotic kanamycin (normally used between 20 and 50 μg mL^−1^). The inhibition and bactericidal assays were also carried out with the eGFP-Mel (melittin at the C-terminus of eGFP) and Mel-eGFP (melittin at the N-terminus of eGFP), which showed a bactericidal effect at 76.80 μg mL^−1^ and 72.10 μg mL^−1^, respectively (Fig. 1 from the Supplementary Material). The Mel-CgDEF performed nearly identically when compared with the peptides without the fusion with CgDEF, in which the AMPs fused to eGFP were only 1.67× more potent than the BiFuProt harboring the peptide anchor CgDEF. CgDEF originates from *Crassostrea gigas* and shows a cystine stabilized-α-β-motif, which comprises of a helical structure and two β-strands cross-linked by three to four disulfide bonds (Gueguen et al. 2006). The anchor CgDEF, fused to eGFP in eGFP-CgDEF, was inactive against *X. citri* (Fig. 1 from the Supplementary Material). Therefore, Melittin is indeed the responsible for the anti-*X. citri* activity displayed by the BiFuProt Mel-CgDEF.

Stover et al. (2013) studied the effects of antimicrobial peptides against *X. citri* and observed that melittin at 1 mM exhibited the lowest minimum inhibitory concentration (MIC) among the diverse AMPs that were investigated. Our findings in the present work not only corroborate the bactericidal action of melittin (in the form of the BiFuProt Mel-CgDEF) against *X. citri*, but it also shows that it targets primarily the bacterial membrane of this plant pathogen. Results of fluorescence microscopy also showed that the peptide does not interfere with cell division or Z-ring formation in *X*. *citri* cells, which is a novel result for melittin.

Through the results from greenhouse assays, it was possible to affirm that Mel-CgDEF could efficiently protect the orange leaves against *X*. *citri* infection, with all settings designed to simulate the infection of citrus trees in the field. The success of melittin in protecting citrus is probably correlated to its capacity of inhibiting and dissolving the biofilm of microorganisms (Picoli et al. 2017). One of the main steps for the success of *X*. *citri* infection is the ability to fixate on the citrus leaf-surface and subsequent formation of bacterial biofilms, which precedes bacterial entry into the leaf mesophyll (Ference et al. 2018; Li et al. 2021). Therefore, the capacity of the BiFuProt to target the bacterial cell membrane, inhibit and/or break biofilm formation is probably taking place at the first stages of infection on the citrus leaf surface. The protection level obtained with Mel-CgDEF was comparable to a copper-based formulation that constitutes the main class of bactericides used for citrus protection (Behlau et al. 2017).

However, the protection profile of the BiFuProt Mel-CgDEF in the assays, in which a washing step was included to simulate rain, was diminished twofold in comparison with the copper formulation. After the washing step, leaves covered with the copper formulation exhibited practically the same protection profile as those not subjected to the washings. Both sets of leaves treated with copper showed a reduction of nearly 90% in the number of lesion per leaf area (from 17.63 lesions/cm^2^, control untreated, to 1.92 lesions/cm^2^, treated with copper). On the other hand, Mel-CgDEF was less retained after the washing step and showed a reduction of 50% in the number of lesion per leaf area (from 17.63 lesions/cm^2^ in the control untreated to 8.95 lesions/cm^2^ in plants treated with the BiFuProt). The copper oxychloride that is the main compound of Difere® is less polar than Mel-CgDEF, consequently showing also a strong adhesion even after washing. Our BiFuProt formulation does not contain, in contrast to commercial preparations, any spread-enhancing or surfactant compounds. In addition, it is worth mentioning that the assay described above was carried out in a greenhouse with controlled parameters of temperature and humidity. In the field, copper is applied several times during the season and always following an event of rain, which is believed to ensure protection effectiveness. The application of copper in citriculture is extensive and frequent (Lamichhane et al. 2018), generating environmental impacts on soil, water, and human health (Coelho et al. 2020). The possibility of having an alternative that can reduce the amount of copper applied in the field is a high advantage to citriculture, and surely to other cultures (Tóth et al. 2016; Lamichhane et al. 2018; Coelho et al. 2020). BiFuProt such as Mel-CgDEF can therefore, from our point of view, be regarded as enabling technology for sustainable plant health management since it is not harmful to humans and the environment due to its not cumulative and biodegradable nature. In addition to being an alternative to copper, the application of tailor-made BiFuProt could be done together or alternated with copper, thus reducing the applied amount of this hazardous metal in the environment.

In conclusion, the BiFuProt produced in the present study is bactericidal against *X*. *citri* at 125 μg mL^−1^, targeting the cytoplasmic membrane on the first minutes of contact. By using a *X*. *citri* mutant strain labelled for the divisional septum, we showed that Mel-CgDEF does not target cell division. BiFuProt can also efficiently protect citrus leaves against *X*. *citri* infection at 250 μg mL^−1^. Although the protective effect is reduced after rain, the BiFuProt is still a novel and possible alternative to copper application on citrus orchards, being able to decrease the frequent amount of applied copper in the field. The rain fastness of the BiFuProts might be further increased via protein engineering and the implementation of adjuvants within the formulation. Furthermore, the developed BiFuProt can be used in situations of low incidence of citrus canker to completely avoid the usage of copper. With increasing incidences of the disease, the BiFuProt might be combined or replaced by copper applications. Before we can recommend growers to use the BiFuProt, the activity and efficacy of BiFuProts have to be studied under field conditions.

## Supplementary information

ESM 1 (PDF 356 kb)

## Data Availability

All the data produced in this work are stored at the online drive server from the research group. The server is not public, but when requested the link and key access will be shared for the access.
