# Supplementary material for: Bifunctional peptides as alternatives to copper-based formulations to control citrus canker
Source: Appl Microbiol Biotechnol. 2024 Feb 7;108:196. doi: 10.1007/s00253-023-12908-3 (PMC10850181; doi:10.1007/s00253-023-12908-3)
Supplement: Supplementary file 1 [file 253_2023_12908_MOESM1_ESM.pdf]

Applied Microbiology and Biotechnology

Supplementary Materials

Bifunctional peptides as alternatives to copper-based formulations to control citrus canker

Guilherme Dilarri[1,*], Leticia Celia de Lencastre Novaes[2,*], Felix Jakob[2], Ulrich Schwaneberg[2,3,+], Henrique Ferreira[4,+]

[1]Department of Fisheries Engineering and Biological Sciences, Santa Catarina State University (UDESC), Rua Coronel Fernandes Martins 270, Postal code: 88790-000, Laguna-SC, Brazil.

[2]DWI – Leibniz-Institute for Interactive Materials, Forckenbeckstraße 50, Postal code: 52056, Aachen, Germany.

[3]Institute of Biotechnology, RWTH Aachen University, Worringerweg 3, Postal code: 52074, Aachen, Germany.

[4]Institute of Biosciences, Biochemistry Building, Department of General and Applied Biology, State University of Sao Paulo (UNESP), Avenida 24-A 1515, Postal code: 13506-900, Rio Claro-SP, Brazil.

G. Dilarri: guilherme.dilarri@udesc.br – ORCID: 0000-0003-2625-7392

L. C. L. Novaes: lclnovaes@gmail.com – ORCID: 0000-0003-4488-2239

F. Jakob: jakob@dwi.rwth-aachen.de – ORCID: 0000-0002-9815-2066

U. Schwaneberg: u.schwaneberg@biotec.rwth-aachen.de – ORCID: 0000-0003-4026-701X

H. Ferreira: henrique.ferreira@linacre.oxon.org – ORCID: 0000-0002-9183-9420

* Authors contributed equally to this work (co-first authorship)

+ Correspondence

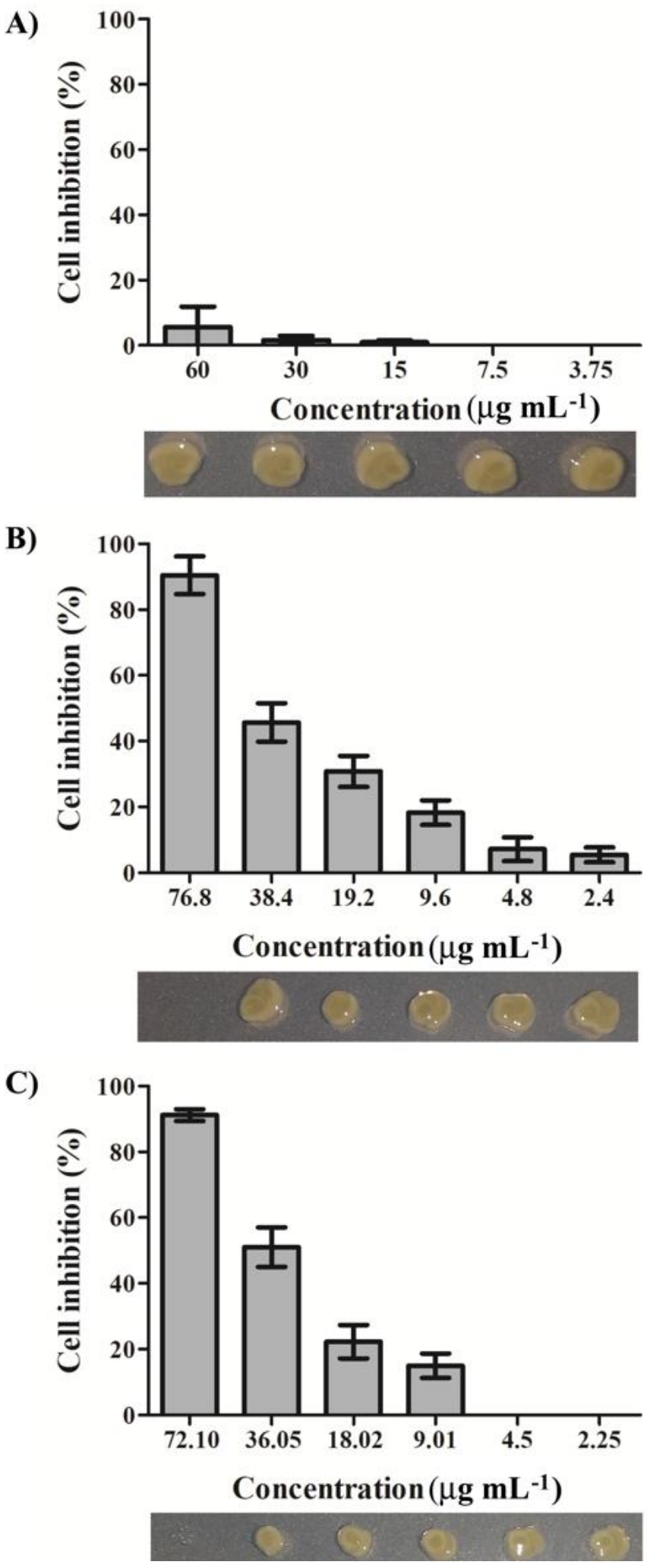

Fig. S1. Evaluating the antibacterial action of the BiFuProt modules used to construct Mel-CgDEF. The respiratory activity of *X. citri* cells was monitored by REMA following 16 hours of exposure to the BiFuProt modules at various concentrations. **A)** eGFP-CgDEF; **B)** eGFP-Mel, and **C)** Mel-eGFP. The average percentages of cells inhibited by the peptide are represented as bars and the standard deviation of the means by the vertical lines above the bars. Underneath the graph is shown a section of a NYG-plate in which samples from REMA were inoculated in order to evaluate if the BiFuProt modules had bactericidal or bacteriostatic effect. Yellow cell mass (colonies) indicate growth.

Table S1. Amino acid sequences, PDB and UniProt entries for all the peptides used.

Peptide	Abbr.	Amino acid sequence (from N- to C-terminus)	PDB	UniProt
Androctonin	Andt	RSVCRQIKICRRRGGCYYKCTNRPY	1CZ6	P56684
LCI	LCI	AIKLVQSPNGNFAASFVLDGTKWIFKSKYYDSSKGYWVGIYEVWDRK	2B9K	P82243
Plantaricin A	PlnA	KSSAYSLQMGATAIKQVKKLFKKWGW	1YTR	P80214
Tachystatin A2	TA2	YSRCQLQGFNCVVRSYGLPTIPCCRGLTCRSYFPGSTYGRCQRY	1CIX	Q9U8X3
Defensin	CgDEF	GFGCPGNQLKCNNHCKSISCRAGYCDAATLWLRCTCTDCNGKK	2B68	Q4GWV4
MBP-1	MPB1	RSGRGECRRQCLRRHEGQPWETQECMRRCRRRG		P28794
Melittin	Mel	GIGAVLKVLTTGLPALISWIKRKRQQ	2MLT	P01501

Table S2. Amino acid and DNA sequence of the bifunctional peptide and its five elements: melittin, 3xGGGS, StrepII-tag, domain Z, and CgDEF (from N- to C-terminus).

	**N-Melittin-**	**-3xGGGS-**	**-StrepII-tag-**	**-Domain Z-**	**- CgDEF -C**
AA	MGIGAVLKVLT<br>TGLPALISWIKR<br>KRQQ	GGGGSG<br>GGGSGG<br>GGS	SAWSHPQF<br>EK	ADNKFNKEQQNAFYEILHLPNLNEEQ<br>RNGFIQSLKDDPSQSANLLAEAKKLN<br>DAQAPK	GFGCPGNQL<br>KCNNHCKSIS<br>CRAGYCDAA<br>TLWLRCTCT<br>DCNGKK
DNA	ATGGGAATTG<br>GTGCTGTGTTG<br>AAGGTTCTTAC<br>TACCGGCCTGC<br>CAGCGTTAATT<br>TCCTGGATTAA<br>ACGTAAGCGG<br>CAA	CAGGGT<br>GGTGGTG<br>GTAGTGG<br>TGGCGGT<br>GGTTCAG<br>GCGGTG<br>GCGGTA<br>GC	TCTGCATG<br>GAGCCATC<br>CGCAGTTC<br>GAAAAG	GCCGACAACAAGTTTAACAAAGAA<br>CAGCAGAACGCCTTCTATGAAATTC<br>TGCATCTGCCGAATCTGAATGAAGA<br>ACAGCGTAATGGTTTTATCCAGAGC<br>CTGAAAGATGATCCGAGCCAGAGC<br>GCAAATCTGCTGGCCGAAGCAAAA<br>AAACTGAATGATGCGCAGGCACCG<br>AAA	GGTTTTGGT<br>TGTCCGGGT<br>AATCAGCTG<br>AAATGTAAC<br>AATCATTGC<br>AAAAGCATT<br>AGCTGCCGT<br>GCAGGTTAT<br>TGTGATGCA<br>GCAACCCTG<br>TGGCTGCGT<br>TGTACCTGT<br>ACCGATTGT<br>AATGGCAAA<br>AAA

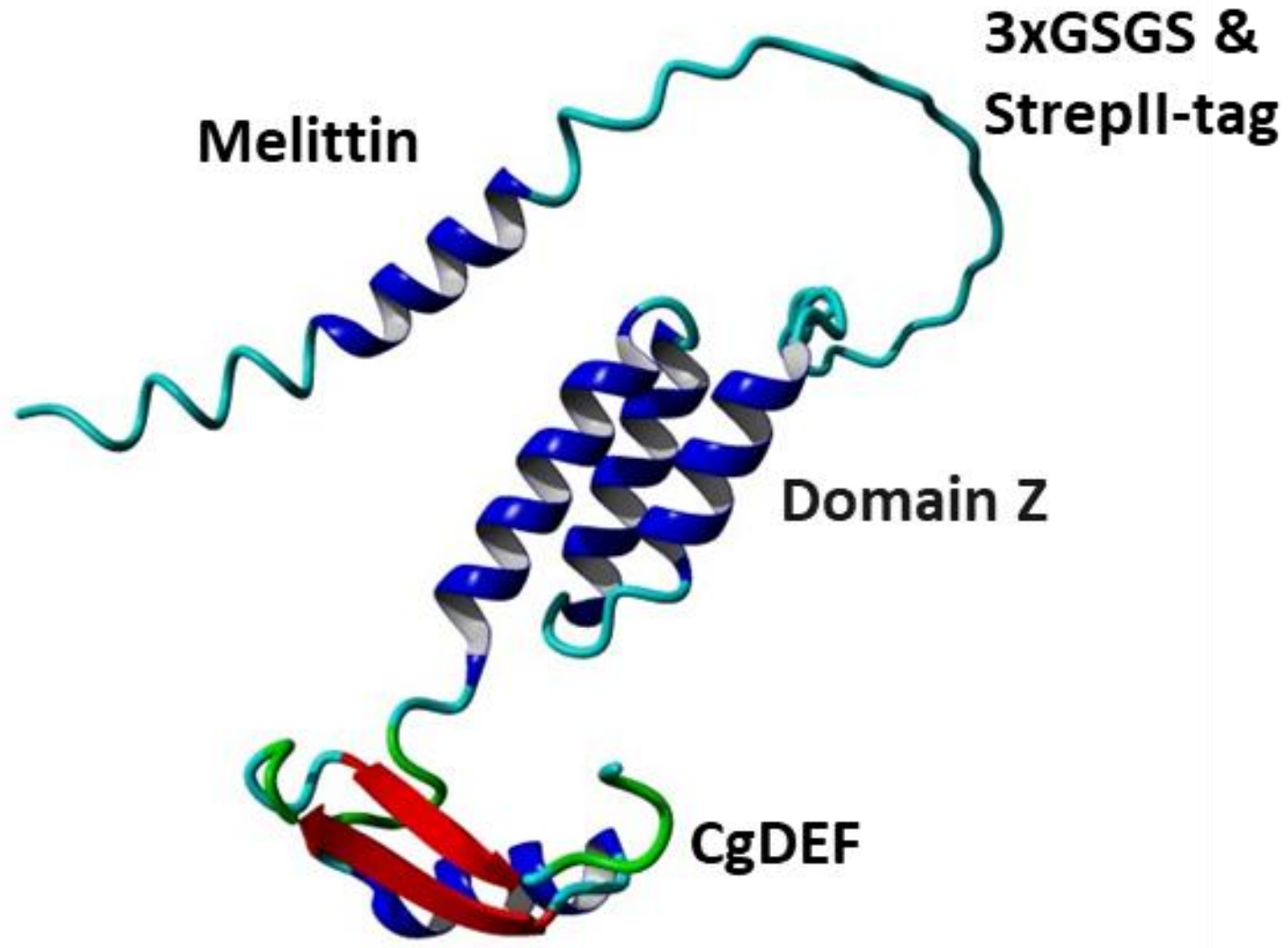

Fig. S2. 3D Protein Structure Model of the BiFuProt Mel-CgDEF. The model was generated using the established AlphaFold 2.0[1] structure database using the amino acid sequence from Tab. S2. The best ranked model is shown.

[1] Jumper J, Evans R, Pritzel A, Green T, Figurnov M, Ronneberger O, Tunyasuvunakool K, Bates R, Žídek A, Potapenko A, et al. (2021) Highly accurate protein structure prediction with AlphaFold. Nature 596:583–589.
